# Is a Repeat Head CT Scan Necessary After an Initial Negative Scan in Head Trauma Patients on DOAC Therapy in the Emergency Department?

**DOI:** 10.3390/jcm15197465

**Published:** 2026-09-25

**Authors:** Emad Talhami, Laith Khader, Shaden Salameh

**Affiliations:** 1 Department of Emergency Medicine, Hadassah Mount Scopus Hospital, Jerusalem 91240, Israel; 2 Faculty of Medicine, Hadassah Hebrew University, Jerusalem 91120, Israel

**Keywords:** CT scan, head trauma, DOAC, emergency department

## Abstract

**Background**: Head trauma in patients receiving direct oral anticoagulants (DOACs) presents a clinical dilemma because of concern for delayed intracranial hemorrhage (ICH). Although some emergency departments (EDs) routinely perform repeat head CT after an initially negative scan, the clinical value of this practice remains uncertain. **Methods**: We conducted a retrospective observational cohort study at Hadassah Mount Scopus University Hospital Emergency Department (ED), a level 3 trauma center with approximately 70,000 visits per year, spanning January 2019 to December 2022. Adult patients receiving DOAC therapy who sustained blunt head trauma and underwent both an initial and repeat head CT according to institutional protocol were included. Demographic characteristics, clinical presentation, type of DOAC, and CT findings were recorded. Exclusion criteria included: penetrating trauma, multi trauma, identified intracranial hemorrhage on the first scan, age < 18 years, and pregnancy status. **Results**: A total of 421 patients met the inclusion criteria. The mean age was 80.1 years, and 236 patients (56%) were female. Apixaban was the most prescribed DOAC (364 patients, 86%), followed by rivaroxaban (39 patients, 9.5%) and dabigatran (18 patients, 4.5%). Glasgow Coma Scale was 15 in 399 patients (94.7%) on presentation. Five patients (1.2%,95% CI: 0.5–2.8%) had delayed ICH on repeat CT after an initially negative scan. All five patients were older than 75 years and had visible evidence of blunt head trauma, and one required neurosurgical intervention. **Conclusions**: Delayed ICH after blunt head trauma in DOAC-treated patients with an initially negative CT was uncommon. These findings support that routine repeat CT scanning for all patients may be unnecessary, and a more focused subsequent CT scan strategy could ultimately optimize hospital resources without compromising patient safety—specifically among elderly patients, those with a low GCS, or showing evidence of trauma. Future research should also evaluate whether emerging blood-based biomarkers can help identify DOAC-treated patients at risk for delayed intracranial hemorrhage despite an initially negative CT scan.

## 1. Introduction

Traumatic brain injury (TBI) remains a leading cause of morbidity and mortality worldwide, especially in the elderly. In 2014, 39% of patients with trauma in the United States were aged 55 years or older and accounted for more than half of the trauma-related deaths in this population [1]. Falls are the primary mechanism of injury, being responsible for nearly 46% of the trauma-related deaths in people older than 65 years [2].

There is an increasing proportion of patients with TBI who are taking antithrombotic therapy, including warfarin, antiplatelet agents, and more recently, direct oral anticoagulants (DOACs). Although anticoagulation decreases the risk of thromboembolism, it complicates the management of intracranial hemorrhage (ICH), even in minor head trauma [3]. Patients on warfarin with head injury and a negative initial CT have traditionally been observed in the hospital for a period, with or without repeat imaging [4]. The management of patients on DOACs is less well defined; there is no INR when monitoring TBI, and reversal agents are expensive and not always available [5].

The utility of repeat CT scans in this population continues to be debated. Some emergency departments (EDs) recommend performing a repeat CT scan 12–24 h after an initial negative CT scan, while others would prefer that merely clinically observation [6]. Multiple studies have shown that routine repeat imaging in a patient without new neurological deterioration has negligible additional value of any sort and that rates of delayed ICH are exceptionally low [7,8]. As of now, however, there is still no firm consensus, and many institutions remain wedded to a practice of extensive use of resources and protocols.

In recent years, several studies have examined the occurrence of delayed ICH in patients using anticoagulant agents after blunt head trauma. Chenoweth et al. [7] performed a multi-center prospective cohort study with 859 patients and reported low incidence of delayed ICH in patients on anticoagulants or antiplatelet agents, which was like the incidence reported in patients not receiving these medications. Additionally, Puzio et al. [9] completed a systematic review and meta-analysis that reported a low incidence of d-ICH with patients treated with DOACs and suggested repeat CT in these patients may be optional.

On the other hand, older literature on patients receiving warfarin reported delayed ICH at higher rates, with some cases requiring neurosurgery [4]. For DOACs, the literature indicates a safety profile that shows lower rates of intracranial bleeding [10,11,12,13]. The prior studies quoted indicate the importance of separating DOACs from older vitamin K antagonists in creating imaging protocols for imaging of head trauma.

Over the last several years, there have been multiple studies with high quality and large size examining delayed ICH in patients receiving anticoagulants, particularly DOACs. Capsoni et al. [14] performed a multi-center cohort study and found an incidence of d-ICH of 1.8% (95% CI: 1.1–3.0%) in patients treated with DOACs compared to 2.6% in patients on Vitamin K antagonists. Most importantly, most of the cases associated with DOACs were mild and managed non-operatively.

Pan et al. [15] investigated (TBI) outcomes of patients with prior anticoagulation and showed that while anticoagulation was associated with increased in-hospital mortality, there was a low risk of clinically significant delayed ICH. Moreover, a cohort from Ontario [16] (2022) reported that delayed ICH occurred in 1.0% of DOAC patients and 1.8% of warfarin patients in 90 days, with no significant difference in delayed ICH in untreated patients.

Multiple other studies supported this finding. Bergenfeldt et al. [17] (2023) found delayed ICH in only 0.25% of their patients, with no cases requiring surgery; meanwhile, André et al. [18] (2024) showed detecting clinically significant outcomes, like mortality or need for neurosurgery, was exceptionally rare in anticoagulated patients. These newer studies support earlier meta-analyses, though now in the DOAC era they even refine estimates of risk.

Nevertheless, practice patterns differ, related to medicolegal concerns and institutional tradition. CT scanning is overused; it increases the cost of care and length of stay as well as exposes patients to unnecessary radiation. However, in most cases, either warrants consideration of additional predictive factors for risk of a rare, but clinically significant, delayed hemorrhage.

In light of these uncertainties, this study intends to evaluate the value, using real-world evidence from the Hadassah Mount Scopus Emergency Department, of repeat head CT scans for patients who are on DOAC therapy, after a previously negative head CT.

## 2. Materials and Methods

This was a retrospective observational cohort study conducted at the Hadassah Mount Scopus Emergency Department, Jerusalem, Israel. The study was approved in the acknowledgement of the Institutional Review Board (IRB 0079-20-HMO). Waivers of informed consent were received due to the retrospective nature of the review. This study is reported in accordance with the STROBE (Strengthening the Reporting of Observational Studies in Epidemiology) guidelines for observational studies; a completed STROBE checklist is provided as Supplementary Materials.

All consecutive adult patients (≥18 years of age) with blunt head trauma while on DOAC therapy (apixaban, rivaroxaban, or dabigatran) were eligible for inclusion over a four-year period (January 2019–December 2022).

Inclusion criteria:

- Age ≥18 years.
- Documented use of DOACs prior to injury.
- Blunt head trauma with an initial CT scan followed by a repeat CT scan within 24 h as per institutional protocol.

Exclusion criteria:

- Penetrating head injuries.
- Multi trauma or associated life-threatening injuries.
- Patients undergo neurosurgical intervention based on initial CT findings.
- Patients who died before a second CT could be obtained. Patients discharged after the initial negative CT. Consequently, the analytic cohort represents patients who underwent repeat CT and should not be interpreted as all DOAC-treated patients presenting with blunt head trauma.

Data were extracted from the institutional trauma registry and electronic medical records. Variables collected included demographics, Glasgow Coma Scale (GCS) at arrival, type of DOAC prescribed, presence of visible trauma, results of initial and repeat CT scans, and neurosurgical intervention. Radiologist blinding the initial CT could not be confirmed retrospectively because imaging was interpreted as part of routine clinical care.

The primary outcome was delayed ICH identified on repeat CT scan when the initial study was negative for any acute intracranial bleeding. The analysis included demographic and clinical characteristics of patients with delayed ICH.

Descriptive statistics were used to summarize the data. Continuous variables were reported as means plus or minus standard deviations, and categorical variables reported as frequency and percentages. Given the small number of delayed ICH events (*n* = 5), no inferential statistical comparisons or multivariable risk-factor analyses were performed between patients with and without delayed ICH. Observed characteristics were therefore described as clinical observations rather than predictors. Analyses were completed using RStudio, version 2023.01.1.

## 3. Results

Over the study period, 421 patients met the inclusion criteria. The mean age was 80.1 years. Of those, 236 (56%) were female and 185 (44%) were male (Table 1). Apixaban was the most prescribed DOAC used (364 patients, 86%), followed by rivaroxaban (39 patients, 9.5%) and dabigatran (18 patients, 4.5%). Most patients presented with a GCS of 15 (399 patients, 94.7%) while 22 patients (5.3%) had GCS in the 11–14 range.

Among the 421 patients, only five patients (1.2%, 95% CI: 0.5–2.8%) experienced delayed ICH noted on the repeat CT scan after having initially provided a negative result. All five patients were over the age of 75 years and presented with visible signs of trauma. Four were treated with apixaban and one was treated with rivaroxaban. Four patients had a GCS of 15 and one patient had a GCS of 14. Only one of the patients underwent neurosurgical intervention (Table 2).

## 4. Discussion

In this retrospective analysis involving 421 individuals undergoing DOAC therapy who experienced blunt head injuries, only five patients (1.2%) developed delayed ICH on repeat CT after an initial negative scan. All patients with delayed ICH were older than 75 years and had visible signs of trauma; one patient required neurosurgical intervention. These findings suggest that delayed ICH was uncommon in this selected cohort, while clinically significant events may still occur.

Our findings are consistent with the recent literature reporting low rates of delayed ICH among anticoagulated patients after blunt head trauma. Capsoni et al. reported a 1.8% rate of delayed ICH in patients on DOACs, and the Ontario cohort reported a 1.0% incidence. Chenoweth et al. similarly found that delayed ICH in anticoagulated patients was uncommon, while Puzio et al. reported lower delayed ICH rates in patients receiving DOACs than in those receiving warfarin.

This body of evidence supports the interpretation that delayed ICH after an initially negative CT is rare in DOAC-treated patients, although differences in admission criteria, repeat imaging protocols, and follow-up duration should be considered when comparing studies.

Importantly, one delayed ICH case in our study required neurosurgical intervention. This finding emphasizes that although delayed ICH was uncommon, clinically significant deterioration remains possible and supports continued clinical observation and individualized decision-making. The differences may be attributed to the pharmacological characteristics of direct acting oral anticoagulants (DOACs). DOACs have shorter half-lives, predictable pharmacodynamics, and less food or drug interaction compared to warfarin [10,11]. Reversal agents are becoming more prevalent, yet the high cost limits their use in standard practice [12].

From a practical perspective, our study provides insight into the potentially limited yield of routine repeat CT scanning in this selected population. Routine repeat imaging after a negative scan not only increases healthcare costs, but also leads to prolonged hospital stay, excess utilization of resources, and exposure of patients to unnecessary radiation; however, our study did not include a formal health-economics analysis, and the financial implications of repeat CT scanning warrant dedicated evaluation in future work [13]. Given the low rate of delayed ICH in this selected cohort, future prospective studies should evaluate whether repeat CT imaging can be guided by clinical features such as advanced age, reduced GCS, visible trauma, neurological symptoms, renal function, concomitant antiplatelet therapy, and comorbidity burden.

The findings of this study add to a growing literature base supporting a more selective approach to repeat neuroimaging in anticoagulated head trauma patients. Traditional clinical decision instruments for minor head injury, such as the Canadian CT Head Rule, were originally derived and validated in patients not receiving anticoagulant or antiplatelet therapy, and current guidance therefore recommends an initial CT scan for virtually all anticoagulated patients regardless of other clinical findings [19]. A recent prospective observational study specifically examined the performance of the Canadian CT Head Rule in patients on anticoagulant or antiplatelet therapy and found that its high-risk criteria retained meaningful discriminatory value even in this population, suggesting that structured clinical variables, rather than anticoagulant status alone, could eventually help determine which patients require imaging after minor head trauma [19]. Although this instrument has not yet been formally validated for the decision to obtain a repeat CT scan, our results, showing that all five delayed ICH cases occurred in patients older than 75 years with visible external signs of trauma, suggest that similar clinical variables, namely advanced age, depressed GCS, and evidence of trauma, may be useful candidates for future prospective risk-stratification research. These observations suggest that clinical variables such as advanced age, depressed GCS, and visible external trauma may be useful candidates for future risk-stratification research. This is consistent with recent work highlighting the unique physiological vulnerabilities and distinct risk profiles of older adults with traumatic brain injury, further supporting selective repeat-CT strategies in elderly patients [20,21].

The clinical relevance of detecting delayed ICH is also shaped by the therapeutic options available once bleeding is identified. A systematic review and meta-analysis pooling 1832 patients with DOAC-associated ICH found that specific reversal agents achieved successful anticoagulation reversal in the majority of cases: 77% with four-factor prothrombin complex concentrate, 75% with andexanet alfa, and 82% with idarucizumab, although substantial rates of all-cause mortality (11–26%) and thromboembolic complications (5–14%) were reported across agents [22]. These findings indicate that identifying delayed ICH, even when it occurs, does not necessarily translate into a straightforward or risk-free management pathway. This observation further supports the argument that clinical resources may be better directed toward improving recognition of, and rapid response to, neurological deterioration rather than toward routine repeat imaging of asymptomatic patients, since the marginal benefit of detecting an occult bleed must be weighed against the cost, risk, and limited availability of reversal therapy, an issue particularly relevant in institutions where these agents are not universally stocked.

Beyond the DOAC-specific reversal challenge, our findings should be interpreted within the broader context of CT overuse in the evaluation of minor head trauma. A 2024 systematic review and meta-analysis synthesizing data from multiple international cohorts reported that a substantial proportion of CT scans performed for mild head injury did not meet validated clinical criteria for imaging, highlighting a persistent pattern of low-value imaging use across emergency departments [23]. When applied specifically to the practice of routine repeat scanning in anticoagulated patients, this broader pattern of overuse likely compounds unnecessary radiation exposure, prolongs emergency department and inpatient length of stay, and adds avoidable cost without a proportional increase in the detection of clinically significant, actionable pathology. Taken together with our institutional experience, in which only one of 421 patients ultimately required neurosurgical intervention for a delayed bleed, these data lend further support to reconsidering blanket repeat-CT protocols in favor of individualized, risk-based imaging strategies for patients on DOAC therapy.

Finally, these considerations carry direct implications for emergency department workflow and resource allocation. Reducing unnecessary repeat scanning in low-risk DOAC-treated patients could shorten emergency department boarding times, reduce crowding, and free imaging capacity for patients with higher-acuity indications, without compromising patient safety, provided that appropriate clinical observation and return precautions are maintained for those discharged without a second scan.

In stating this, it is worthwhile to mention that while rare, delayed ICH can happen. Therefore, clinical observation is still warranted, particularly in high-risk populations. Future research involving larger prospective cohorts will assist with further risk stratification and the development of best practices regarding imaging for DOAC patients.

## 5. Limitations

This study has several limitations. First, it has a retrospective single-center design, which may limit generalizability. Second, only patients who underwent a repeat CT were included, while patients discharged after the initial negative CT were excluded. Therefore, the reported incidence should be interpreted as the incidence among patients selected for repeat imaging rather than among all DOAC-treated patients presenting with blunt head trauma. Third, the small number of delayed ICH events (*n* = 5) precluded inferential comparisons, multivariable modeling, and formal identification of independent risk factors. Fourth, several potentially relevant variables, including mechanism of injury, loss of consciousness, renal function, concomitant antiplatelet therapy, comorbidity burden, hemorrhage subtype, hemorrhage size, and discharge disposition, were not consistently available in the retrospective dataset. Fifth, radiologist blinding the initial CT could not be confirmed because imaging interpretation occurred as part of routine clinical care. Sixth, Anti-Xa testing was not performed, limiting assessment of anticoagulant activity and bleeding risk [24,25,26,27]. Lastly, follow-up was limited to the hospital stay, and delayed ICH occurring after discharge may not have been captured.

## 6. Conclusions

The incidence of delayed ICH in DOAC-treated patients with a blunt head injury has a relatively low incidence of delayed ICH (1.2%), and only one case required neurosurgical intervention. With this information, routine repeat CT scanning for all patients may be unnecessary, and a more focused subsequent CT scan strategy could ultimately optimize hospital resources without compromising patient safety—specifically among elderly patients, those with a low GCS, or showing evidence of trauma. Although routine repeat CT scanning may increase costs and resource utilization, our study did not include a formal health-economics analysis, and future research should address this gap.

## Figures and Tables

**Table 1:** Patient demographics and clinical characteristics.

Variable	Value
Total number of patients	421
Mean Age	80.1 years
Sex	
Male	185 (44%)
Female	236 (56%)
Neurological Status	
GCS = 15	399 (94.7%)
GCS 11–14	22 (5.3%)
Type of DOAC	
Apixaban	364 (86%)
Rivaroxaban	39 (9.5%)
Dabigatran	18 (4.5%)

**Table 2:** Characteristics of patients with delayed ICH.

Patient	Age	Sex	DOAC Type	GCS	Visible Trauma Signs	Neurosurgical Intervention
1	78	Male	Apixaban	15	Yes	Yes
2	82	Female	Apixaban	15	Yes	No
3	76	Female	Rivaroxaban	14	Yes	No
4	85	Male	Apixaban	15	Yes	No
5	79	Male	Apixaban	15	Yes	No

## Data Availability

The original contributions presented in this study are included in the article/Supplementary Materials. Further inquiries can be directed to the corresponding author.

## Supplementary Materials

The following supporting information can be downloaded at: https://www.mdpi.com/article/10.3390/jcm15197465/s1. Table S1: STROBE Checklist for Cohort Studies.

## Abbreviations

The following abbreviations are used in this manuscript:

DOAC	Direct Oral Anticoagulant
ICH	Intracranial Hemorrhage
GCS	Glasgow Coma Scale
TBI	Traumatic brain injury (TBI)
