# Supplementary material for: Is a Repeat Head CT Scan Necessary After an Initial Negative Scan in Head Trauma Patients on DOAC Therapy in the Emergency Department?
Source: J Clin Med. 2026 Sep 25;15(19):7465. doi: 10.3390/jcm15197465 (PMC13665955; doi:10.3390/jcm15197465)
Supplement: Supplementary file 1 [file jcm-15-07465-s001.zip › jcm-4461683-supplementary.pdf]

Supplementary Material

STROBE Checklist for Cohort Studies

Manuscript: "Is a Repeat Head CT Scan Necessary After an Initial Negative Scan in Head Trauma Patients on DOAC Therapy in the Emergency Department?"

This checklist maps the manuscript to the Strengthening the Reporting of Observational Studies in Epidemiology (STROBE) recommendations for cohort studies. Locations are identified by manuscript section/subsection. "Not reported" indicates information that is not explicitly provided in the submitted manuscript.

Item	STROBE recommendation	Location in manuscript / response
Title and abstract 1a	Indicate the study design with a commonly used term in the title or abstract.	Abstract: "retrospective observational cohort study." Title does not explicitly state "cohort study."
Title and abstract 1b	Provide an informative and balanced summary of what was done and what was found.	Abstract: Background, Methods, Results, and Conclusions.
Introduction 2	Explain the scientific background and rationale for the investigation.	Introduction, Section 1, paragraphs discussing delayed ICH and repeat CT utility.
Introduction 3	State specific objectives, including any prespecified hypotheses.	Introduction, final paragraph (Section 1): evaluates the value of repeat head CT after an initially negative CT in DOAC-treated patients. A formal hypothesis is not stated.
Methods—study design 4	Present key elements of study design early in the paper.	Abstract Methods and Materials and Methods, Section 2: retrospective observational cohort study.
Methods—setting 5	Describe setting, locations, and relevant dates, including recruitment, exposure, follow-up, and data collection periods.	Abstract and Section 2: Hadassah Mount Scopus ED, Jerusalem; January 2019–December 2022. Follow-up was limited to the hospital stay (Limitations, Section 5).
Methods—participants 6a	Give eligibility criteria and sources/methods of selection of participants.	Section 2: inclusion criteria (age ≥18, DOAC use, blunt head trauma, initial and repeat CT within 24 h) and exclusion criteria.
Methods—participants 6b	For matched studies, give matching criteria and numbers exposed/unexposed.	Not applicable: no matching was used.
Methods—variables 7	Clearly define all outcomes, exposures, predictors, potential confounders, and effect modifiers.	Section 2: primary outcome defined as delayed ICH on repeat CT after a negative initial CT; collected variables include demographics, GCS, DOAC type, visible trauma, CT findings, and neurosurgical intervention. Potential confounders/effect modifiers are not comprehensively prespecified.
Methods—data sources/measurement 8	For each variable of interest, give sources and methods of assessment.	Section 2: trauma registry and electronic medical records; CT findings and clinical variables extracted. Radiologist blinding could not be confirmed retrospectively.
Methods—bias 9	Describe any efforts to address potential sources of bias.	Section 2 and Limitations: retrospective single-center design, selection of patients who underwent repeat CT, lack of confirmed radiologist blinding, and missing variables are acknowledged.
Methods—study size 10	Explain how the study size was arrived at.	Section 2: all consecutive eligible adult patients over January 2019–December 2022; final cohort n=421. No a priori sample-size calculation is reported.
Methods—quantitative variables 11	Explain how quantitative variables were handled in analyses; describe categorization if applicable.	Section 2: continuous variables summarized as means ± SD; categorical variables as frequencies and percentages. Age >75 and GCS categories are presented descriptively.
Methods—statistical methods 12a	Describe all statistical methods, including those used to control for confounding.	Section 2: descriptive statistics using RStudio 2023.01.1; no inferential comparisons or multivariable analyses because only five events occurred.
Methods—statistical methods 12b	Describe any methods used to examine subgroups and interactions.	Not reported; no subgroup or interaction analyses were performed.
Methods—statistical methods 12c	Explain how missing data were addressed.	Not explicitly reported. Limitations state that several variables were not consistently available.
Methods—statistical methods 12d	If applicable, describe analytical methods taking account of sampling strategy.	Not applicable/not reported; no complex sampling strategy was described.
Methods—statistical methods 12e	Describe any sensitivity analyses.	Not reported; no sensitivity analyses were performed or described.
Results—participants 13a	Report numbers of individuals at each stage of study—potentially eligible, examined for eligibility, confirmed eligible, included, and analyzed.	Section 2 and Results: 421 patients met inclusion criteria. A complete screening flow with numbers at each stage is not provided.
Results—participants 13b	Give reasons for non-participation at each stage.	Section 2 lists exclusion criteria; stage-specific counts and reasons are not provided.
Results—participants 13c	Consider use of a flow diagram.	Not included in the submitted manuscript. A participant flow diagram may be added if required.
Results—descriptive data 14a	Give characteristics of study participants and information on exposures and potential confounders.	Results, Table 1: age, sex, GCS, and DOAC type. Other potentially relevant variables were not consistently available.
Results—descriptive data 14b	Indicate number of participants with missing data for each variable of interest.	Not reported in a variable-by-variable format.
Results—outcome data 15	Report numbers of outcome events or summary measures over time.	Results, Section 3 and Table 2: five delayed ICH cases (1.2%, 95% CI 0.5–2.8%); one required neurosurgical intervention.

Results—main results 16a	Give unadjusted estimates and, if applicable, confounder-adjusted estimates and precision (e.g., 95% CI).	Results: delayed ICH 1.2% with 95% CI 0.5–2.8%. No adjusted estimates were generated because of the small number of events.
Results—main results 16b	Report category boundaries when continuous variables were categorized.	Results: GCS=15 versus GCS 11–14; delayed ICH cases described as age >75 years.
Results—main results 16c	Translate relative risk estimates into absolute risk for a meaningful time period.	Absolute frequency and proportion are reported (5/421; 1.2%). No relative risk estimate was calculated.
Results—other analyses 17	Report other analyses done, e.g., subgroup analyses and sensitivity analyses.	No additional analyses reported.
Discussion—key results 18	Summarize key results with reference to study objectives.	Discussion, Section 4, opening paragraphs.
Discussion—limitations 19	Discuss limitations of the study, taking into account sources of potential bias or imprecision.	Limitations, Section 5: retrospective single-center design, selection bias, small event count, missing variables, uncertain blinding, no anti-Xa testing, and limited follow-up.
Discussion—interpretation 20	Give a cautious overall interpretation considering objectives, limitations, multiplicity of analyses, results from similar studies, and other relevant evidence.	Discussion, Section 4: findings are interpreted as uncommon delayed ICH in the selected cohort; clinical observation and individualized decision-making are emphasized.
Discussion—generalisability 21	Discuss the generalisability (external validity) of the study results.	Limitations, Section 5: single-center design may limit generalizability; cohort includes only patients selected for repeat CT.
Other information—funding 22	Give the source of funding and the role of the funders.	Funding statement: no external funding. Role of funders is not applicable.
